# Supplementary material for: Predicting individual differences of fear and cognitive learning and extinction
Source: Nat Commun. 2026 Apr 23;17:3780. doi: 10.1038/s41467-026-71830-0 (PMC13109375; doi:10.1038/s41467-026-71830-0)
Supplement: Supplementary file 2 — Reporting Summary [file 41467_2026_71830_MOESM2_ESM.pdf]

nature portfolio

Corresponding author(s): Carlos A. Gomes

Last updated by author(s): Mar 18, 2026

Reporting Summary

Nature Portfolio wishes to improve the reproducibility of the work that we publish. This form provides structure for consistency and transparency in reporting. For further information on Nature Portfolio policies, see our Editorial Policies and the Editorial Policy Checklist.

Statistics

For all statistical analyses, confirm that the following items are present in the figure legend, table legend, main text, or Methods section.

n/a	Confirmed	
☐	☒	The exact sample size (*n*) for each experimental group/condition, given as a discrete number and unit of measurement
☐	☒	A statement on whether measurements were taken from distinct samples or whether the same sample was measured repeatedly
☐	☒	The statistical test(s) used AND whether they are one- or two-sided<br>*Only common tests should be described solely by name; describe more complex techniques in the Methods section.*
☐	☒	A description of all covariates tested
☐	☒	A description of any assumptions or corrections, such as tests of normality and adjustment for multiple comparisons
☐	☒	A full description of the statistical parameters including central tendency (e.g. means) or other basic estimates (e.g. regression coefficient) AND variation (e.g. standard deviation) or associated estimates of uncertainty (e.g. confidence intervals)
☐	☒	For null hypothesis testing, the test statistic (e.g. *F*, *t*, *r*) with confidence intervals, effect sizes, degrees of freedom and *P* value noted<br>*Give P values as exact values whenever suitable.*
☐	☒	For Bayesian analysis, information on the choice of priors and Markov chain Monte Carlo settings
☐	☒	For hierarchical and complex designs, identification of the appropriate level for tests and full reporting of outcomes
☐	☒	Estimates of effect sizes (e.g. Cohen's *d*, Pearson's *r*), indicating how they were calculated

Our web collection on statistics for biologists contains articles on many of the points above.

Software and code

Policy information about availability of computer code

Data collection	Stimulus presentation software Presentation (Neurobehavioral Systems).<br>Voltage stimulators STM2000 (BIOPAC Systems) and DS7A (Digitimer Ltd).
Data analysis	(f)MRI data were preprocessed using fmriprep (v20.1.1) and MRtrix3 (v3.0.4). In addition, we used FreeSurfer (v7.2), Matlab (v2022b) toolboxes (SPM12, Wavelet Toolbox), FSL (v6.0.6.1), ANTs (v2.2.0) and Python 3 libraries (nibabel, nipype, nilearn, pycwt, dtw-python) for various processing stages. R (4.2.2) libraries (ggplot2, lme4, glmnet, emmeans, dplyr, car, caret, MASS, psych), as well as several Python libraries (numpy, sklearn, pandas, scipy) were used for statistical analyses and data visualisation. EDA data were analysed using the Matlab toolbox PsPM (v6.0.6). Additional custom scripts were written in Python 3, Matlab and R. Please see https://github.com/caadgomes/extinction-learning-study/requirements.txt for a complete list of software/libraries used in this study.<br>The scripts for running all analyses are available at https://github.com/caadgomes/extinction-learning-study

For manuscripts utilizing custom algorithms or software that are central to the research but not yet described in published literature, software must be made available to editors and reviewers. We strongly encourage code deposition in a community repository (e.g. GitHub). See the Nature Portfolio guidelines for submitting code & software for further information.

Data

Policy information about availability of data

All manuscripts must include a data availability statement. This statement should provide the following information, where applicable:

- Accession codes, unique identifiers, or web links for publicly available datasets
- A description of any restrictions on data availability
- For clinical datasets or third party data, please ensure that the statement adheres to our policy

Public sharing of the raw data is not permitted because participant consent and ethics approvals do not allow unrestricted public data sharing, and the datasets are subject to institutional and legal data-sharing agreements across the contributing institutions. Raw data are available from the corresponding author upon reasonable request and subject to approval by the contributing institutions and a data-sharing agreement where required. Access will be granted to qualified researchers for non-commercial scientific research purposes; decisions are typically made within 4–6 weeks. Source data are provided with this paper.

Research involving human participants, their data, or biological material

Policy information about studies with human participants or human data. See also policy information about sex, gender (identity/presentation), and sexual orientation and race, ethnicity and racism.

Reporting on sex and gender	Only sex of participants was recorded. It was not of interest for any of our research questions, and we only included it as a covariate in our models to control for potential confounds.
Reporting on race, ethnicity, or other socially relevant groupings	n/a
Population characteristics	See section "Behavioural & social sciences study design".
Recruitment	Participants were university students who were recruited via university message boards and/or mailing lists. All participants gave written informed consent and were monetarily reimbursed or received course credits.
Ethics oversight	The individual studies contributing to this project were approved by the Ethics Committee of the Medical Faculty of the University of Duisburg-Essen (Ethik-Kommission der Medizinischen Fakultät der Universität Duisburg-Essen) and by the Ethics Committee of the Ruhr University Bochum.

Note that full information on the approval of the study protocol must also be provided in the manuscript.

Field-specific reporting

Please select the one below that is the best fit for your research. If you are not sure, read the appropriate sections before making your selection.

☐ Life sciences ☒ Behavioural & social sciences ☐ Ecological, evolutionary & environmental sciences

For a reference copy of the document with all sections, see nature.com/documents/nr-reporting-summary-flat.pdf

Behavioural & social sciences study design

All studies must disclose on these points even when the disclosure is negative.

Study description	Quantitative experimental
Research sample	Our research sample consisted of university students from the Ruhr University Bochum and from the University of Duisburg-Essen. Our sample is representative and the sample size was chosen on the basis of available data collected from the independent research projects.<br>Resting-state fMRI: S1, N=28 [age = 24.4 (3.51 SD), 19 women]; S2, N=152 [age = 22.0 (2.20 SD), 95 women]; S3, N=44 [age = 23.5 (3.56 SD), 22 women]; S4, N=177 [age = 25.8 (4.19 SD), 89 women]; S5, N=56 [age = 24.1 (3.74 SD), 26 women]; S6, N=56 [age = 26.3 (4.66 SD), 38 women].<br>Difussion MRI: S2, N=166 [age = 21.9 (2.17 SD), 103 women]; S3, N=44 [age = 23.5 (3.56 SD), 22 women]; S4, N=175 [age = 25.7 (4.04 SD), 85 women]; S5, N=56 [age = 24.1 (3.55 SD), 25 women]; S6, N=56 [age = 26.5 (4.85 SD), 27 women].
Sampling strategy	The sample consisted of university students recruited through independent research projects (convenience sampling). Data collection was not blinded to study hypotheses. There was no predertemined sample size, since our study aggregated datasets acquired from several independent research projects.
Data collection	Data collection involved a computer for presenting stimuli and recording participants' responses during the task in the MRI scanner, a specialised system for recording electrodermal activity in the MRI scanner, and pen and paper for questionnaires.<br>Both the researcher of each specific project and the participant were present during the experiment. The researcher was not blind to experimental condition or study hypothesis during data collection.
Timing	August 2017 until September 2021

Data exclusions	For the SCR analyses, 56 acquisition and 79 extinction datasets were excluded due to flat or extremely coarse SCR data (see Fig. S2 for examples of typical excluded datasets).
Non-participation	Given that each research group of the consortium only provided us with the data from participants who participated in the studies, we do not have information on non-participation.
Randomization	Participants were randomly assigned to experimental groups.

Reporting for specific materials, systems and methods

We require information from authors about some types of materials, experimental systems and methods used in many studies. Here, indicate whether each material, system or method listed is relevant to your study. If you are not sure if a list item applies to your research, read the appropriate section before selecting a response.

Materials & experimental systems

n/a	Involved in the study
☒	☐ Antibodies
☒	☐ Eukaryotic cell lines
☒	☐ Palaeontology and archaeology
☒	☐ Animals and other organisms
☒	☐ Clinical data
☒	☐ Dual use research of concern
☒	☐ Plants

Methods

n/a	Involved in the study
☒	☐ ChIP-seq
☒	☐ Flow cytometry
☐	☒ MRI-based neuroimaging

Plants

Seed stocks	*Report on the source of all seed stocks or other plant material used. If applicable, state the seed stock centre and catalogue number. If plant specimens were collected from the field, describe the collection location, date and sampling procedures.*
Novel plant genotypes	*Describe the methods by which all novel plant genotypes were produced. This includes those generated by transgenic approaches, gene editing, chemical/radiation-based mutagenesis and hybridization. For transgenic lines, describe the transformation method, the number of independent lines analyzed and the generation upon which experiments were performed. For gene-edited lines, describe the editor used, the endogenous sequence targeted for editing, the targeting guide RNA sequence (if applicable) and how the editor was applied.*
Authentication	*Describe any authentication procedures for each seed stock used or novel genotype generated. Describe any experiments used to assess the effect of a mutation and, where applicable, how potential secondary effects (e.g. second site T-DNA insertions, mosiacism, off-target gene editing) were examined.*

Magnetic resonance imaging

Experimental design

Design type	resting-state
Design specifications	n/a
Behavioral performance measures	n/a

Acquisition

Imaging type(s)	Functional, structural and diffusion MRI
Field strength	3T
Sequence & imaging parameters	Structural: T1-weighted images (MP-RAGE) with parameters TR = 8 ms, TE = 4 ms, flip angle = 8°, voxel size = 1 x 1 x 1 mm3, FOV = 24 x 24 cm (studies S1,S2,S4,S5); TR = 2.53 ms, TE = 2 ms, flip angle = 7°, voxel size = 1 x 1 x 1 mm3, FOV = 19.2 x 25.6 cm (study S3); TR = 1.77 ms, TE = 3 ms, flip angle = 8°, voxel size = 1 x 1 x 1 mm3, FOV = 19.2 x 25.6 cm (study S6).<br>Functional: T2*-weighted images acquired using a gradient echo, echo-planar imaging (EPI) sequence with the following parameters TR = 2.5 s, TE = 30 ms, flip angle = 90°, voxel size = 3 x 3 x 3 mm3, FOV = 24 x 24 cm, 80 x 80 voxels, number of slices = 47, number of volumes = 190 (studies S1,S2,S4); TR = 1.43 s, TE = 30 ms, acceleration factor = 2, flip angle = 69°, voxel size = 3 x 3 x 3 mm3, FOV = 24 x 24 cm, 80 x 80 voxels, number of slices = 48, number of volumes = 190 (study S3); TR = 2.5 s, TE = 30 ms, flip angle = 90°, voxel size = 3 x 3 x 3 mm3, FOV = 28 x 31 cm, 92 x 92 voxels, number of slices = 46, number of volumes = 192 (study S6).
Area of acquisition	Whole-brain

Diffusion MRI ☒ Used ☐ Not used

Parameters: TR = 9.5 s, TE = 88 ms, flip angle = 90°, voxel size = 2 x 2 x 2 mm3, FOV = 24 x 24 cm, 112 x 112 voxels, number of slices = 60, number of directions = 60 (b = 1000 s/mm2) (studies S1,S2,S4); TR = 5.5 s, TE = 114 ms, flip angle = 90°, voxel size = 1.6 x 1.6 x 1.6 mm3, FOV = 24 x 24 cm, 132 x 128 voxels, number of slices = 60, number of directions = 60 (b = 1000 s/mm2) (study S3); TR = 10.2 s, TE = 87 ms, flip angle = 90°, voxel size = 2 x 2 x 2 mm3, FOV = 60 x 60 cm, 120 x 120 voxels, number of slices = 70, number of directions = 60 (b = 1000 s/mm2) (study S6).

Preprocessing

Preprocessing software: fmriprep (v23.1.4), FSL (v6.0.6.1), MRtrix3 (v3.0.4)

Normalization: Data were not normalised as all of our analyses were done in native space.

Normalization template: Data were not normalised.

Noise and artifact removal: 24 head motion parameters (six base motion parameters + six temporal derivatives of six motion parameters + 12 quadratic terms of six motion parameters and their six temporal derivatives), CSF and WM mean signal, global signals, detrending.

Volume censoring: Volume censoring was not performed.

Statistical modeling & inference

Model type and settings: Multilevel mixed-effects models and regularized regression were used to examine associations between different types of brain connectivity measures and learning efficacy.

Effect(s) tested: We tested the predictive effects of functional, effective, and structural connectivity between predefined ROIs on trial-wise learning indices. Effects corresponded to the association between brain connectivity and learning efficacy during acquisition, extinction, and renewal phases. No factorial ANOVA designs were used; inference was based on mixed-effects modelling and cross-validated regularised regression.

Specify type of analysis: ☐ Whole brain ☒ ROI-based ☐ Both

Anatomical location(s): Anatomical locations were based on Desikan-Killiany atlas (available in FreeSurfer) for the amygdala, hippocampus, ventro-medial prefrontal cortex and anterior cingulate cortex. We used the Matlab toolbox SUIT for the extraction of the cerebellar nuclei.

Statistic type for inference (See Eklund et al. 2016): n/a

Correction: FDR, permutations and Monte-carlo.

Models & analysis

n/a	Involved in the study
☐	☒ Functional and/or effective connectivity
☒	☐ Graph analysis
☐	☒ Multivariate modeling or predictive analysis

Functional and/or effective connectivity: Functional connectivity: Pearson correlation, cross-correlation, dynamic time warping, Euclidean distance, Manhattan distance, Wasserstein distance, mutual information, magnitude-squared coherence, Wavelet coherence.
Effective connectivity: spectral dynamic causal modeling.

Multivariate modeling and predictive analysis: Independent variables were the brain connectivity values. Regularised regression models (LASSO, Ridge, Elastic Net) with 10-fold cross-validation implemented to evaluate model performance.
